# Clinical Uncertainty Influences Antibiotic Prescribing for Upper Respiratory Tract Infections: A Qualitative Study of Township Hospital Physicians and Village Doctors in Rural Shandong Province, China

**DOI:** 10.3390/antibiotics12061027

**Published:** 2023-06-08

**Authors:** Liyan Shen, Ting Wang, Jia Yin, Qiang Sun, Oliver James Dyar

**Affiliations:** 1 Center for Health Management and Policy Research, School of Public Health, Cheeloo College of Medicine, Shandong University, Jinan 250012, China; 2 NHC Key Lab of Health Economics and Policy Research (Shandong University), Jinan 250012, China; 3 Department of Public Health and Caring Sciences, Uppsala University, 75122 Uppsala, Sweden

**Keywords:** clinical uncertainty, antibiotic use, primary care, China

## Abstract

Objective: This study aimed to explore how clinical uncertainty influences antibiotic prescribing practices among township hospital physicians and village doctors in rural Shandong Province, China. Methods: Qualitative semi-structured interviews were conducted with 30 township hospital physicians and 6 village doctors from rural Shandong Province, China. A multi-stage random sampling method was used to identify respondents. Conceptual content analysis together with Colaizzi’s method were used to generate qualitative codes and identify themes. Results: Three final thematic categories emerged during the data analysis: (1) Incidence and treatment of Upper Respiratory Tract Infections (URTIs) in township hospitals and village clinics; (2) Antibiotic prescribing practices based on the clinical experience of clinicians; (3) Influence of clinical uncertainty on antibiotic prescribing. Respondents from both township hospitals and village clinics reported that URTIs were the most common reason for antibiotic prescriptions at their facilities and that clinical uncertainty appears to be an important driver for the overuse of antibiotics for URTIs. Clinical uncertainty was primarily due to: (1) Diagnostic uncertainty (establishing a relevant diagnosis is hindered by limited diagnostic resources and capacities, as well as limited willingness of patients to pay for investigations), and (2) Insufficient prognostic evidence. As a consequence of the clinical uncertainty caused by both diagnostic and prognostic uncertainty, respondents stated that antibiotics are frequently prescribed for URTIs to prevent both prolonged courses or recurrence of the disease, as well as clinical worsening, hospital admission, or complications. Conclusion: Our study suggests that clinical uncertainty is a key driver for the overuse and misuse of prescribing antibiotics for URTIs in both rural township hospitals and village clinics in Shandong province, China, and that interventions to reduce clinical uncertainty may help minimize the unnecessary use of antibiotics in these settings. Interventions that use clinical rules to identify patients at low risk of complications or hospitalization may be more feasible in the near-future than laboratory-based interventions aimed at reducing diagnostic uncertainty.

## 1. Introduction

Antibiotic resistance is seriously affecting global public health, with predictions forecasting grave impacts both in terms of mortality and morbidity, and economically, if concerted and coordinated actions are not taken [1]. Since the inappropriate use of antibiotics unnecessarily accelerates antibiotic resistance, reducing the overuse and misuse of antibiotics is a key component in efforts to manage the threat posed by antibiotic resistance.

In terms of the total annual consumption of antibiotics by country, China is now second [2]. The average annual consumption of antibiotics for each Chinese person has previously been reported as 138 g, which is 10 times that consumed in the United States [3]. Surveillance studies have tracked the emergence and spread of many drug-resistant bacterial strains in China over the past few decades, and it is clear that what occurs in China will have large implications for global patterns of antibiotic resistance spread [4]. 

Since the 1960s, a three-tier healthcare system has existed in urban and rural areas in China. Many policies have been issued in China for improving the use of antibiotics over the past fifteen years. However, these policies have been largely targeted toward reducing the use of antibiotics in secondary and tertiary hospitals [5]. Recent studies show that antibiotic prescribing rates remain high in primary healthcare settings (consisting of urban community health centers and stations, as well as rural township hospitals and village clinics), with higher rates of inappropriate and unnecessary antibiotic prescribing, particularly in rural areas [6,7,8]. There are variations in the training of clinicians working at different rural primary healthcare institutions. For instance, township hospitals are staffed by a mixture of licensed physicians (who typically have a five-year bachelor’s degree or higher and majored in medicine at university), as well as many assistant licensed physicians (who are typically graduates of universities or junior college and who typically hold a three-year medical vocational degree). In contrast, village clinics are staffed by “village doctors,” typically with a single village doctor per clinic. The title of “village doctor” has been used by the Chinese government since 1985 to replace the previous title of “Barefoot doctor”. Village doctors are permitted by local authorities to work in rural village clinics if they hold a village doctor certificate instead of a regular medical license [9]. Qualifications for receiving a village doctor certificate include either (1) A technical school education, including training in basic public health services and diagnosis and management of common diseases in rural areas (about 3 years), or (2) Over 20 years’ experience of clinical practice in rural clinics.

Previously, studies have estimated that over 90% of patient consultations with Upper Respiratory Tract Infections (URTIs) at township hospitals result in an antibiotic prescription [10]; these prescriptions are usually unnecessary, however, given that most UTRIs are viral diseases and self-limiting in nature [11]. There are complex reasons for the overuse and misuse of antibiotics in rural primary care settings in China, including associations with limitations in clinicians’ knowledge about antibiotics and antibiotic resistance [12,13], inappropriate attitudes among clinicians towards antibiotic prescribing [14], patient expectations and demands for clinicians to prescribe antibiotics [15], and inappropriate fiscal incentives [12,16]. In addition to these factors, increasing attention is being paid to the role that clinical uncertainty may have in driving antibiotic overuse and misuse. 

Clinical uncertainty is an intrinsic part of clinical practice, which can be manifested in processes ranging from diagnostic and therapeutic decisions to understanding prognoses [17]. This uncertainty can broadly be defined as a cognitive state characterized by an awareness of the incomplete understanding of a situation or event [18]. In the context of infections of uncertain etiology (i.e., diagnostic uncertainty), a prescription of antibiotics may provide a form of protection for patients and clinicians by appearing to minimize uncertainty surrounding prognosis (i.e., if the infection proved to be bacterial, then hopefully the prescribed antibiotics will improve prognosis) [19]. More broadly, it has been suggested that inappropriate antibiotic use can be driven by different aspects of clinical uncertainty, including diagnostic uncertainty [20], lack of prognostic evidence [1,21], and limitations in physician and patient tolerance of diagnostic uncertainty [22]. A study conducted by Xue et al. with simulated patients in rural areas of three provinces in China suggested that diagnostic uncertainty is a far more significant contributor to unnecessary antibiotic prescribing than knowledge gaps among clinicians [21]. A separate study in the Hubei province found that primary care physicians in both rural and urban areas experienced a high frequency of clinical uncertainty, which resulted in more intense antibiotic use, particularly for URTIs [23]. Clinical uncertainty likely affects primary care physicians in rural China disproportionately due, in part, to resource limitations [21,23,24].

Significant research on clinical uncertainty in other settings worldwide has been conducted, strongly indicating that clinical uncertainty is associated with higher levels of antibiotic prescribing [1,21,25]. The specific reasons for clinical uncertainty are often context- and culture-specific, and understanding how clinical uncertainty influences clinician practices concerning antibiotic use in rural China is currently limited. Thus, this study aims to explore how clinical uncertainty influences the practice of antibiotic prescribing among physicians working at township hospitals, as well as among village doctors working in village clinics, using rural Shandong Province as a case study. The study also investigates clinicians’ perceptions concerning the potential ways in which clinical uncertainty could be reduced in these settings.

## 2. Materials and Methods

### 2.1. Study Design and Setting

This qualitative research study consisted of face-to-face semi-structured interviews with township hospital physicians and village doctors in rural Shandong Province. Shandong Province, in eastern China, reportedly had 100.5 million inhabitants in 2020, making it the second largest province in China in terms of population [26]. Rural areas in the province share many similarities with other rural areas in eastern China regarding the population’s education levels, health indicators, and per-capita income.

A multi-stage sampling method was used in a three-step process. First, 4 administrative cities (an administrative unit similar to ‘counties’ or ‘regions’ in other countries) were selected from the 17 administrative cities in the Shandong Province to account for variation in terms economic development level (high, medium, and low) and geographic location (western, eastern, and central). Secondly, for each administrative city, 2 township hospitals in rural areas were carefully selected; these township hospitals were visited by the research team, and 3–4 physicians who were available on the day of interviews were invited to participate. For every selected township hospital, one village clinic under the administrative jurisdiction of the township hospital was also selected; the village doctor working at the clinic on the day of data collection was invited to participate.

The principle of data saturation was used to determine study sample size, with interviews taking place until no new themes or subthemes emerged [27]. In this study, saturation was reached after interviews with the 28th township hospital physicians and 5th village doctors, respectively, with additional respondents failing to provide new information. Additionally, 3 extra respondents (2 township hospital physicians and 1 village doctor) were interviewed to ensure data saturation, and data collection stopped after the 36th respondent was interviewed. The interviews took place between 10 June 2021 and 23 July 2021 at 9 township hospitals and 6 village clinics, with data saturation appearing after interviews at the 8th township hospital and 5th village clinic.

### 2.2. Sampling

The inclusion criteria for physicians at township hospitals included the following: at least 5 years of professional experience in township hospitals; self-report of a relevant practice qualification (board certification) in pediatrics or general practice; and being a full-time physician primarily practicing in an outpatient office setting. The inclusion criteria for village doctors in village clinics included the following: holding a village doctor certificate; at least 5 years of practice in village clinics.

In total, 36 interviews were completed with 30 township hospital physicians and 6 village doctors.

### 2.3. Interview Guide and Data Collection

The semi-structured interview guide (Supplementary Material S1) was developed by experts in clinical medicine and public health and was informed by previous studies [21,28,29,30]. The interview guide covered demographic information, the incidence and treatment of URTIs in primary healthcare settings, practices of antibiotic prescribing, and influences of clinical uncertainty on antibiotic prescribing. The two primary authors conducted the semi-structured interviews, with one responsible for interviewing clinicians and the other recording the interviews and managing time. To ensure the interviews were of high quality, the two researchers were trained in qualitative research and conducted several simulation exercises before the formal interviews. 

All interviews were conducted in a quiet room at the healthcare facility and were audio-recorded. The interviews lasted an average of 30 min (ranging from 25 to 41 min). Open-ended questions guided the interviews and interviewers followed-up responses with prompts for further detail when necessary. Each respondent who completed the interview received financial compensation for lost work (RMB 100; approx. USD 15.6).

### 2.4. Data Analysis

An iFLYTEK software program (version SR502, iFLYTEK Co., Ltd., Shenzhen, China) was used to store the recorded interviews to protect the privacy of respondents. The recorded interviews were transcribed into text, which was then assessed by two researchers to ensure accuracy, and thereafter imported into NVivo (version 10). Two researchers led the analysis of the data in order to minimize subjective bias and errors, in accordance with conceptual content analysis [31] and Colaizzi’s [32,33] method. There were 7 steps to the data analysis: (1) Two researchers read each transcript several times to understand the entirety of the interview; (2) Two researchers critically read and reviewed the transcript of each respondent’s answers, highlighting sentences and phrases directly associated with the research objectives. A consensus was achieved after comparing the work of the two researchers; (3) Researchers coded the meanings formulated in important statements into various categories; (4) Grouping of categories was conducted based on themes, where thematic clusters associated with a specific issue formed a new theme; (5) Each theme was described in detail. A third, senior researcher with a deep knowledge of the study field reviewed the findings to confirm if the descriptions appeared to accurately reflect how clinical uncertainty influences the antibiotic prescribing practices of township hospital physicians and village doctors; (6) After the review, descriptions were modified for clarity; (7) The researchers shared the fundamental structure statement with study respondents and 5 experts (3 from public health and 2 from clinical medicine) to confirm if it was capable of accurately reflecting their wider experiences in antibiotic prescribing practices in rural China, particularly in Shandong province. We report the results following the consolidated criteria for reporting qualitative research (COREQ) checklist (Supplementary Material S2) [34].

## 3. Results

### 3.1. Demographic Characteristics

The demographic characteristics of the respondents are shown in Table 1. Of the 30 physicians working in township hospitals, 63% (19/30) were male and 77% (23/30) had worked in healthcare for 11 or more years. A total of 83% (25/30) had obtained a general practice qualification and 17% (5/30) had obtained a medical pediatrics qualification. Of the six village doctors, only one was female, and 84% (5/6) had worked in healthcare for 11 or more years. All village doctors had received high school- or technical school-level education.

### 3.2. Thematic Categories

Three final thematic categories emerged during the data analysis (as described above): (1) incidence and treatment of URTIs in township hospitals and village clinics; (2) antibiotic prescribing practices based on the clinical experience of clinicians; (3) influence of clinical uncertainty on antibiotic prescribing. These thematic categories are described in more detail in Section 3.3, Section 3.4 and Section 3.5.

### 3.3. Incidence and Treatment of URTIs in Township Hospitals and Village Clinics

The township hospital physicians and village doctors described the common diseases suffered by patients in their respective primary healthcare settings. Due to the aging rural population and migration of working-age adults to cities, there are fewer young adults in rural areas; thus, most of the villages are populated by empty-nest elderly people or left-behind children. Consequently, chronic diseases account for a significant proportion of the diseases managed by primary healthcare facilities in these areas (both township hospitals and village clinics), and these chronic diseases include hypertension, diabetes, and chronic bronchitis.

“*Nowadays, there are mostly older people and children in the village, and few young people*.”

(P25, township hospital physician)

Village doctors and township hospital physicians reported that URTIs characterized by the common cold, fever, and cough are common diseases among patients visiting both village clinics and township hospitals. However, the proportion of visits to village clinics that can be attributed to URTIs is usually higher than at township hospitals.

“*Generally, in addition to chronic disease, upper respiratory tract infections (URTIs) are a common disease at the primary healthcare settings*.”

(P31, township hospital physician)

“*Compared with township hospitals, upper respiratory tract infections (URTIs) are more common in village clinics, accounting for 60 to 70% (visits). It (township hospital) can account for one-third…probably less than one-half (for patients with URTIs)*.”

(P12, township hospital physician)

“*Most (residents) in village are elderly and children, and there are (among elderly people) usually more chronic diseases like hypertension and diabetes. In addition, there are more patients with the common cold, (symptoms) such as fever, cough, and runny nose*.”

(P06, village doctor)

### 3.4. Antibiotic Prescribing Practices Based on the Clinical Experience of Clinicians

This thematic category included five sub-themes, all of which emerged in interviews with respondents from both township hospitals and village clinics:

***(1)*** *Antibiotics are prescribed according to patients’ symptoms.* Overall, 27 (90%) physicians said that, when a patient has symptoms that they considered to be of suspected bacterial infection, such as tonsil suppuration, pharyngeal congestion, rhinorrhea and sputum, antibiotics are prescribed to the patient. Village doctors expressed the same opinion.

“*I prescribe mainly based on clinical symptoms and clinical experience. If the patient has symptoms (of suspected bacterial infections), such as high fever, tonsil suppuration, and runny nose, antibiotics are also used*.”

(P25, township hospital physician)

“*I usually treat symptomatically and use antibiotics if the patient has symptoms of bacterial infection, such as purulent rhinorrhea, coughing up yellow sputum*.”

(P03, village doctor)

***(2)*** *Antibiotics are ideally prescribed according to medical investigations but are in practice based largely on clinical experience.* At township hospitals, with better infrastructure and resources, physicians reported that they would preferably first recommend laboratory tests for patients with URTIs (such as routine blood tests and C-reactive protein), and then prescribe medicines based on a combination of test results and patients’ symptoms. However, in rural areas, patients will often refuse tests for reasons such as being unable to afford the tests, or a lack of time. Therefore, in practice, many township hospital physicians prescribe drugs based on their clinical experience and the patient’s symptoms. The most common antibiotics prescribed for URTIs are Cephalosporins and Azithromycin. Respondents from village clinics acknowledged that laboratory tests could be helpful, but reported that diagnostic resources are extremely limited, thus forcing them to rely on clinical experience.

“*Sometimes patients don’t listen to you. For example, if you ask them to check the blood first to see (what the condition is), they will refuse on the grounds of ‘high price’, ‘too busy, no time’, and ‘too many people’*.”

(P16, township hospital physician)

“*We can only rely on clinical experience. Cephalosporin and Azithromycin are the commonly used antibiotics prescribed for patients*.”

(P15, township hospital physician)

“*Because I don’t have any tools (to aid decision-making), antibiotics can only be prescribed based on clinical experience*.”

(P29, village doctor)

***(3)*** *Special consideration for chronic diseases.* Respondents from both township hospitals and village clinics reported that the common presence of chronic diseases and additional comorbidities among patients is one of the main justifications for being more likely to prescribe antibiotics, with the rationale being that it is more important to minimize the chances of an infection becoming severe in such patients.

“*Older people have more chronic diseases and antibiotics are often used prophylactically*.”

(P01, township hospital physician)

“*Generally, some elderly people are not in good health. Most of them have chronic diseases, such as bronchitis, high blood pressure, heart disease and chronic obstructive pulmonary disease. These patients will usually use antibiotics prophylactically even if they have no symptoms (of suspected of bacterial infection). Antibiotics are always used to prevent infection from getting worse*.”

(P07, village doctor)

***(4)*** *Special consideration for the time of year.* Respondents reported that antibiotics are prescribed more often during busy farming seasons, with many township hospital physicians mentioning that injectable antibiotics are used more frequently at this time of year. Village doctors reported that patients want an instant cure, especially in busy farming seasons, and this can lead to a higher rate of antibiotic use. If patients are unable to recover quickly and are delayed in their ability to work, then there can easily be economic losses that will affect their financial security for the next year.

“*Especially in the summer, it is the busy agricultural season, and there are many patients who inject antibiotics*.”

(P09, township hospital physician)

“*Antibiotics are generally used to reduce inflammation. If patients are not prescribed antibiotics, they will not be able to recover in time*.”

(P29, village doctor)

“*Although it is a common cold, due to the busy season of farming, patients cannot recover in time if antibiotics are not used. If patients delay work because of their illness, all melons will rot in the rural land, and the whole family will have to drink the wind (in the next year*).”

(P29, village doctor)

***(5)*** *Strong influence of patients’ expectations and demands.* Both township hospital physicians and village doctors reported that patients have strong expectations and demands for antibiotics when they have URTI symptoms and that these are important driving factors for the prescribing of antibiotics. Failing to meet patient demands can lead to negative consequences for the doctor. For instance, potential consequences include complaints from the patient being forwarded to a higher authority, and loss of potential income if the patient chooses to visit another healthcare provider instead in the future. A number of township hospital physicians and village doctors reported feeling pressured to prescribe antibiotics due to potential impacts on their reputation and a belief that they need to have a similar willingness to prescribe antibiotics as their other colleagues have.

“*Most of the patients know that they have common cold, and when they come here (township hospital), they will say ‘I want to take some antibiotics’ or ‘Please give me an intravenous injection. Every time when I have a cold, I have to do this to get better’*.”

(P20, township hospital physician)

“*If the patient’s expectations are not met, complaints will be met. At this time, the director will ask (me) if there is any refusal to prescribe the drug. If it is not handled well, it will be punished*.”

(P33, township hospital physician)

“*There is peer pressure. In the current environment, we cannot be alone, because patients cannot be kept without antibiotics. When another doctor prescribed antibiotics to meet the patient’s expectation, I lost a patient*.”

(P16, township hospital physician)

“*Patients want an instant cure so they try a doctor’s medicine for a day or two, and don’t want to delay working to make money. If they don’t recover for a long time, they may seek consultation with another doctor*.”

(P23, township hospital physician)

*As far as I am concerned, 80%–90% of patients (with common cold) will use antibiotics*.

(P07, village doctor)

“*If other doctors prescribe antibiotics, but I do not give antibiotics to the patients, then the patients who come to see me will always be unable to recover for a long time, and gradually no patients come to me*.”

(P29, village doctor)

### 3.5. Influence of Clinical Uncertainty on Antibiotic Prescribing

Two themes are included within this thematic category: *(1) Sources of clinical uncertainty* and *(2) Risk factors in predicting patient outcomes.*

#### 3.5.1. Sources of Clinical Uncertainty

Two sub-themes emerged among the responses of interviewees:

***(1)*** *Diagnostic uncertainty contributes to clinical uncertainty.* As alluded to earlier, resource limitations in terms of diagnostic capabilities exist at both township hospitals and village clinics, as does an unwillingness of patients to undergo tests they perceive to be expensive or time-consuming. Twenty-three (73%) township hospital physicians made it clear that they understood that most URTIs are of a self-limiting viral etiology. However, they still choose to prescribe antibiotics, partially because of diagnostic uncertainty and a consequential defensive prescribing practice in order to minimize clinical uncertainty.

“*There are limited (diagnostic) facilities at primary care facilities, so it is not possible to directly determine whether URTIs are bacterial or viral. Like such as county (secondary) and city (tertiary) hospitals, where people can directly do routine blood tests or C-reactive protein, and doctors there have confidence in making decisions. It’s not like here (primary care facilities), where it’s all about fear and responsibility. It’s really hard to be a doctor these days…*”

(P17, township hospital physician)

“*Village clinics are the most poorly equipped with infrastructure, lower than township hospitals*.”

(P06, village doctor)

“*About 80%–90% of URTIs are viral diseases, which are of self-limiting nature. We all learned it when we went to school. But seeing a patient is not a class, you can’t follow the textbook, and no patient gets sick according to knowledge in the textbook. So, in order to avoid unnecessary troubles, more antibiotics will be prescribed to prevent worsening*.”

(P25, township hospital physician)

***(2)*** *Lack of prognostic evidence contributes to clinical uncertainty.* Twenty-three (77%) township hospital physicians and five (83%) village doctors mentioned that, although URTIs are usually viral infections, bacterial infections can occur afterwards; therefore, there are risks if antibiotics are not prescribed prophylactically. Consequently, both township hospital physicians and village doctors reported that antibiotics are often prescribed “just in case” to reduce the likelihood of either a prolonged course/recurrence of the disease, clinical worsening, hospital admission, or complications (e.g., pneumonia). Respondents from both township hospitals and village clinics reported the fear that if they employed a watch-and-wait policy or prescribed only rehydration solution packets for fever, patients may not recover as quickly and could be in pain or discomfort for longer periods.

“*If antibiotics are not used, it may cause worsening or prolong the course of the disease. Patients can recover in 3 days, but without prescribing antibiotics, they may not be able to recover in 10 days.*”

(P18, township hospital physician)

“*When antibiotics are not used in time, it will lead to repeated attacks and even worsening of the disease*.”

(P03, village doctor)

“*It can cause complications, such as bacterial infections, pneumonia, acute bronchitis, or other diseases*.”

(P10, township hospital physician)

“*It can cause complications such as rhinitis, pharyngitis, urinary tract infection, acute bronchitis, pneumonia, etc*.”

(P03, village doctor)

#### 3.5.2. Factors in Predicting Patient Outcomes

Respondents were asked about factors that could predict patient outcomes (i.e., potential prognostic indicators) to help reduce the clinical uncertainty in cases of URTI. Township hospital physicians and village doctors reported a range of factors that they believed could provide early warnings of a more severe illness (Table 2). Three main aspects were described, again by both township hospital physicians and village doctors: 

***(1)*** *Clinical symptoms*. When the patient has symptoms that are perceived to be compatible with bacterial infection (such as sore throat, cough, yellow sputum, tonsil suppuration) the disease can become worse if antibiotics are not used in time.

“*If symptoms such as sore throat, cough with yellow sputum, hyperemia in throat, and pharyngeal congestion occur, it is usually a bacterial infection. If antibiotics are not used, the condition may worsen*.”

(P20, township hospital physician)

*“I usually… use antibiotics if the patient has symptoms of bacterial infection, such as purulent rhinorrhea, coughing up yellow sputum*.”

(P03, village doctor)

***(2)*** *Comorbidities and previous illnesses.* Doctors stressed that comorbidities, age, and a personal history of pneumonia are risk factors that lead to worse outcomes from an URTI.

“*Patients with chronic bronchitis and heart disease generally have poor immunity. If you don’t use antibiotics, patients’ conditions can easily get worse*.”

(P9, township hospital physician)

“*Patients with a history of pneumonia and tumors are more prone to complications*.”

(P15, township hospital physician)

“*Most of them have chronic diseases, such as bronchitis, high blood pressure, heart disease and chronic obstructive pulmonary disease… Antibiotics are always used to prevent the disease from getting worse*.”

(P07, village doctor)

***(3)*** *Seasonal factors.* Township hospital physicians reported that winter and summer are the seasons wherein there is a higher incidence of URTIs and that these infections result in worse outcomes in these seasons.

“*Generally, when the temperature is very low, especially in winter, it is not easy for the patient to recover. If antibiotics are not used, it is easy to get serious*.”

(P15, township hospital physician)

“*In summer or when the rural land is busy, it is easy to fall ill. If antibiotics are not prescribed to patients to reduce inflammation, uncontrollable things may occur. For example, the patient has a sudden shock or suffocation, which has happened before*.”

(P19, township hospital physician)

## 4. Discussion

Our findings suggest that antibiotic overuse and misuse for URTIs remain common in both township hospitals and village clinics in rural Shandong province, despite recent policy strategies involving both restrictive and persuasive interventions [4,35]. In 2012, the Chinese Ministry of Health issued a regulation to limit the antibiotic prescription rate to 20% of outpatient prescriptions in all patients. This has not been very successful in rural areas, as evidenced by a study in the Guanxi province in 2017, which identified that 68% of outpatient pediatric visits to township hospitals for URTIs resulted in antibiotic prescription [36]. Our own previous surveillance studies at village clinics in the Shandong province over a 2.5-year period found an overall antibiotic prescription rate of 40.3%, with 62.5% of visits for URTIs resulting in antibiotic prescription [8]. 

In keeping with previous qualitative studies in rural China [12,37], respondents in the present study reported that antibiotic prescribing practices were based primarily on patients’ symptoms rather than the results of medical investigations (irrespective of facility type) and that specific consideration is given to the presence of comorbidities and the time of year. Respondents at both township hospitals and village clinics further mentioned the strong influence of patient expectations and demands as well as the negative consequences of failing to meet these.

We found a notable lack of variation in the responses between township hospital physicians and village doctors, despite the differences between township hospitals and village clinics in terms of the educational background of clinicians and the availability of diagnostic resources. Although potentially surprising, this lack of variation is in keeping with a previous survey study we conducted in rural Shandong province, in which we found very similar patterns of knowledge and attitudes towards antibiotic prescribing among township hospital physicians and village doctors [29]. We have no single explanation for these observed similarities, but we suggest here four inter-related factors that may be relevant. First, both healthcare facility types serve patient populations with similar socio-demographic backgrounds and clinical cases (i.e., URTIs among elderly patients account for a large proportion of visits at both of these types of rural primary healthcare facility). Second, patients at both facility types appear to have very similar expectations and demands, and these appear to be significant in shaping clinicians’ practices (e.g., prescribing antibiotics to maintain a client or to avoid loss of reputation and/or income). Third, clinicians at both village clinics and township hospitals appear to exhibit a similar lack of clinical knowledge that could impact on diagnostic and prognostic uncertainty (e.g., only two of thirty township hospital physicians, and no village doctors whatsoever, mentioned specific lung auscultation findings as a risk factor for more severe disease [pneumonia] in the context of URTIs, whereas less discriminatory signs and symptoms [rhinorrhea, fever, and pharyngeal redness] were commonly mentioned). Finally, although additional diagnostic resources are available at township hospitals, their use is limited in practice by a lack of willingness among patients, which means that clinicians at both types of health facility have similar levels of diagnostic information to base their recommendations on. In summary, although there may be differences between township hospital physicians and village doctors in terms of background education and the availability of diagnostic resources, in practice, they may face very similar clinical situations on a daily basis, with similar levels of relevant clinical knowledge, similar levels of clinical uncertainty, and similar pressures from patients, which, ultimately, all shape their behaviors.

The present study also provides new and context-specific insights into the role that clinical uncertainty has in shaping antibiotic prescribing decisions among clinicians in rural Shandong province, suggesting that it may be an important driver of unnecessary antibiotic prescriptions. Respondents reported that URTIs were the most common reason for patients visiting township hospitals and village clinics, which is in line with previous studies in rural China [38,39], and that URTIs are the major diagnosis for which antibiotics are prescribed. Although the vast majority of URTIs are self-limiting, meaning it is usually possible to ‘wait and see’, few respondents reported adopting the strategy [25]. Respondents reported that, due to diagnostic and prognostic uncertainty, they prescribe antibiotics just in case, aiming to mitigate the perceived risk of complications (such as hospital admission).

The concept of uncertainty avoidance has been described in Hofstede’s model of cultural dimensions [40]. People feel threatened by uncertainty, ambiguity, and an uncertain future, and the extent to which they try to minimize uncertainty varies between cultures and societies [40]. As previous studies have reported, uncertainty avoidance is relevant to medical situations, and patients and physicians are expected to have an aversion to clinical uncertainty and to prefer a clear prognostic evidence-base for decision-making [41]. Therefore, a low tolerance of uncertainty among physicians can generally lead to the over-prescribing of antibiotics [25,42] since physicians are unwilling to accept prognostic uncertainty and, consequently, prescribe antibiotics to mitigate uncertainty somewhat [29,38]. In the context of diagnostic and prognostic uncertainty, patients’ demands and expectations may have a more significant role to play in shaping the behaviors of a clinician, since the clinician is less able to simply reassure a patient. Our findings suggest that the situation is being further exacerbated in practice by knowledge deficits among both clinicians and patients. For instance, common misunderstandings identified in this study include the idea that antibiotics can reduce inflammation irrespective of the underlying cause, and that antibiotics can still reduce recovery time in the case of viral illnesses, and these findings are in keeping with previous qualitative work in rural China [12,37]. Beyond the cultural dimension of uncertainty avoidance, other recent work has also highlighted potential associations between patterns of antibiotic consumption within a country and the broader cultural values of the country. For instance, the Inglehart–Welzel cultural map of the world categorizes countries along two dimensions: traditional versus secular-rational values and survival versus self-expression values. China lies in the “Confucian” cluster of countries and regions, many other of which also have relatively high antibiotic consumption levels (e.g., Taiwan, South Korea) [42].

Many respondents from both village clinics and township hospitals mentioned that if they could identify patients with a low risk of suffering complications, then they felt that they could reduce antibiotic use for these patients. This finding is in line with results from recent international studies, which are beginning to provide evidence that reducing physicians’ clinical uncertainty in practice can reduce antibiotic prescription rates in primary care [23,43]. Interventions that can reduce clinical uncertainty among individual patients may help to further reduce unnecessary antibiotic use alongside other antibiotic stewardship interventions (e.g., education, prescription audits, regulations), whose impact has been more limited thus far in rural China [44]. In this context, important technological developments have occurred in recent years—aimed at reducing diagnostic uncertainty. For example, a recent trial in Vietnam showed that C-reactive protein testing was capable of reducing antibiotic use for non-severe acute respiratory tract infections in primary healthcare settings [45]. Similarly, recent studies have shown that procalcitonin may have a role to play in reducing diagnostic uncertainty, particularly in inpatient settings, but potentially even in low-risk outpatient settings [46,47]. However, there are important limitations regarding improving diagnostic capacity in both township hospitals and village clinics in China [48]. The relatively high cost of C-reactive protein testing is currently prohibitive, and more generally, laboratory confirmation and testing for antibiotic sensitivity are restricted in most public health facilities. Furthermore, as indicated by many respondents in the present study, patients are typically required to pay for the diagnostic services; thus, they must be willing to do so (i.e., perceive sufficient value). Together, these limitations in diagnostic capacity result in clinicians being heavily reliant on their clinical acumen, and approaches to reduce clinical uncertainty in rural China centered solely on improving diagnostic capacity may not ultimately have high levels of feasibility in the near-future.

As well as efforts to reduce diagnostic uncertainty, there may also be promise in efforts aimed at reducing prognostic uncertainty, such as the development, implementation, and use of guidelines and clinical treatment pathways. These may be capable of identifying patients who are at the highest risk of experiencing complications (for example, by stratifying patients according to risk of future hospital admission for URTIs) [1]. Our study found that respondents from both township hospitals and village clinics were generally aware that the prognosis for URTIs can be affected by a range of factors (e.g., severity of clinical symptoms, existence of comorbidities), even if there may be lack of knowledge concerning the relative prognostic significance of different risk factors. There may be an important role for future research studies to develop and validate context-specific clinical rules that can identify patients with URTIs who are most at risk of complications, potentially drawing on developments in big data and machine learning [49,50].

In addition to reducing clinical uncertainty, many respondents from both township hospitals and village clinics suggested other ways of improving antibiotic use in rural primary care settings in China. Many physicians suggested that better regulation would be one of the more effective measures to promote the rational use of antibiotics, particularly improving the supervision of village clinics in rural areas. Such supervision has been historically neglected in comparison with the monitoring of antibiotic use in hospitals and in urban areas, even though prescriptions in village clinics account for a significant proportion of total antibiotic use in China. The use of software capable of monitoring and giving feedback to clinicians at village clinics on the rational use of antibiotics in near-real-time was found to improve antibiotic prescribing in a study in Anhui province [51]. Developing and, crucially, implementing such solutions for village clinics more widely in rural China could also help promote rational antibiotic use. Finally, many township hospital physicians and village doctors mentioned the importance of patient education to help address the issue of patient demand for antibiotics, implying that it would be beneficial for patients to learn the key differences between anti-inflammatory medicines and antibiotics as well as the importance of using antibiotics only when they are clinically necessary. In this context, it may even be worth emphasizing the increasing certainty around the negative effects of antibiotic use (e.g., on a patient’s microbiome) in contrast to the uncertainty of their beneficial effects in the vast majority of URTI illnesses.

### Study Strengths and Limitations, and Future Directions

Our study has several strengths, such as the involvement of researchers with considerable experience in the subject area and the methodological stage in which the fundamental structure statement was discussed with study respondents and external experts, both of which contribute to the credibility and confirmability of the study’s findings. Our study also has several limitations. First, the study was conducted only in Shandong Province, China. Although the study selected representative rural areas of the province by considering economic development level (high, medium and low) and the geographic location, respondents ultimately came from an extremely small proportion of total healthcare facilities in Shandong province. Furthermore, the study had a small number of total respondents, particularly in terms of the number of village doctors, although data saturation was reached. Our study findings likely have limited generalizability beyond Shandong province, although certain results may be relevant for other rural areas in Eastern China due to similarities in population as well as in the healthcare system structure and its personnel. Since China has quite a unique healthcare system (for instance, due to its historical development), the transfer or application of our findings to other countries, even with similar levels of socio-economic development, should be performed with caution. For instance, it is beyond the scope of this study to directly compare the study area with areas in which significantly lower antibiotic prescription rates are commonly found (e.g., primary care institutions in many European countries such as Sweden or the Netherlands). Longstanding antibiotic stewardship programs have been present in many such areas, combining a range of different interventions over time (e.g., clinical guidelines, rapid diagnostic tests, prescriber education, use of anti-inflammatory medications). It would nonetheless be interesting for future studies to assess the ways in which clinical uncertainty is managed in these areas, and indeed in lower-prescribing areas in China, to facilitate a comparison with the prescribing rates in rural Shandong province. A final limitation worth noting is that, due to the broad and exploratory nature of our study, we focused on the decision to prescribe antibiotics and did not attempt to investigate in more detail the appropriateness of dosing and duration; it may be worthwhile for future studies to consider if and how these aspects are affected by clinical uncertainty.

## 5. Conclusions

Township hospital physicians and village doctors in rural Shandong province, China, reported commonly prescribing antibiotics unnecessarily. Our findings suggest that clinical uncertainty is an important and under-investigated driver of unnecessary antibiotic prescriptions for URTIs, both in township hospitals and village clinics. Clinicians commonly engage in defensive antibiotic prescribing in order to mitigate uncertainty concerning diagnosis and prognosis, with the expectations and demands of patients further increasing the pressure on clinicians to prescribe antibiotics. Interventions to reduce clinical uncertainty may be able to reduce antibiotic misuse and overuse, and interventions that use clinical rules to identify patients at low risk of complications or hospitalization may be more feasible in the near-future than laboratory-based interventions aimed at reducing diagnostic uncertainty. Such interventions should be considered alongside the on-going implementation of other antibiotic stewardship measures.

## Figures and Tables

**Table 1:** Descriptive statistics of the study sample.

Characteristic	Number of Township Hospital Physicians (N = 30)	Percent	Number of Village Doctors (N = 6)	Percent
*Sex*				
Male	19	63	5	83
Female	11	37	1	17
*Age (in years)*				
30-39	12	40	-	-
40-49	12	40	4	67
50-59	6	20	2	33
*Working experience (in years)*				
5–10	7	23	1	17
11–20	11	37	1	17
21–30	7	23	3	50
31–40	5	17	1	17
*Highest Education Level*				
Junior high school or below	1	3	-	-
High school or technical school	10	33	6	100
Undergraduate or above	19	63	-	-
*Practice qualification*				
General practice	25	83	-	-
Medical pediatrics	5	17	-	-
Village doctor certificate	-	-	6	100

**Table 2:** Factors that increase the likelihood of severe illness with URTI symptoms, as reported by township hospital physicians and village doctors.

		Frequency of Reported Terms *	
Dimension	Sub-Dimension	Township Hospital Physicians (N = 30)	Village Doctors (N = 6)
Clinical symptoms and course of the illness	Cough with yellow/green sputum	30	6
	Hyperemia/redness in throat	30	6
	Pus on the tonsils	30	6
	Fever	26	4
	Rhinorrhea (yellow/green discharge)	22	4
	Sore throat	19	4
	Cough without sputum	15	5
	Headache	12	3
	Sneeze	11	1
	Overall course of the illness	6	1
	Abnormal lung auscultation	2	0
Comorbidities and previous illnesses	Diabetes	26	4
	Hypertension	20	5
	Chronic obstructive pulmonary disease	19	3
	Chronic bronchitis	17	2
	History of pneumonia	15	2
	Heart disease	14	1
	Old age	12	0
	History of a tumor	10	0
Seasonal factor	Season (temperature)	7	3

* Refers to how many township hospital physicians or village doctors mentioned this sub-dimension.

## Data Availability

All data generated or analyzed during this study are included in the tables.

## Supplementary Materials

The following supporting information can be downloaded at: https://www.mdpi.com/article/10.3390/antibiotics12061027/s1, S1: The semi-structured interview guide; S2: COREQ checklist.

## Abbreviations

URTIs: upper respiratory tract infections; COREQ: criteria for reporting qualitative research.
