# Supplementary material for: Clinical Uncertainty Influences Antibiotic Prescribing for Upper Respiratory Tract Infections: A Qualitative Study of Township Hospital Physicians and Village Doctors in Rural Shandong Province, China
Source: Antibiotics (Basel). 2023 Jun 8;12(6):1027. doi: 10.3390/antibiotics12061027 (PMC10295071; doi:10.3390/antibiotics12061027)
Supplement: Supplementary file 1 [file antibiotics-12-01027-s001.zip › Supplementary Material 1.pdf]

Supplementary Materials 1: Topic guide for primary care physicians

Date:__________________ Settings(specific name): _________________

1. Introduction and explain the nature and purpose of the study

Thank you for agreeing to take part in the interview. Our study aims to explore how the practice of antibiotic prescription. We aim to explore how the clinical uncertainty influences practice of antibiotic prescription for physicians and how to reduce clinical uncertainty in future study. The information you provide will help us improve our understanding of practice of antibiotic prescription and our intervention refinement.

2. Confirm consent to take part

I will start by audio recording your consent to take part in this interview – I will turn my audio recorder on now and ask you the consent questions – please answer yes or no.

1) Do you agree to our conversation being audio recorded?
2) Do you know you are free to stop the interview at any point and you may skip questions you would prefer not to answer?
3) Do you understand that when we write about our research, we may quote what you said but it will be anonymous and not be possible to identify you?
4) Do you understand that we will keep a record of this interview for future research but without anything that could identify you?

3. Topic guide

Part 1: Demographic Information

1. Sex? Male; Female
2. Age
3. Work year

4. Department in township hospital or village clinic
5. Highest Education Level? Junior high school or below; High school or technical school; Undergraduate or above
6. Practice qualification? General practice; Medical pediatrics; Practice qualifications of rural doctors

Part 2: Research Questions

1. Can you talk about the situation of medical service in primary care facilities?
2. Could you please describe the common diseases in primary care facilities? What is your treatment measure for the common diseases? (The commonly used medicines and the commonly used antibiotics)
3. Could you please describe the circumstances in which you prescribe antibiotics?
4. What are the factors that influence your antibiotic prescription? What was particularly important about factors and why?
5. Could you please describe the impacts of the clinical uncertainty during prescribing antibiotics?
6. What do you think is an effective way to promote the rational use of antibiotics?
7. Is there anything you'd like to add about prescribing antibiotic that is important from your opinion?

4. End

1. It is entirely voluntary to take part in this research. You are free to withdraw at any time without providing any reason, and this will not have any impact on your work.
2. All information is confidential. No name or personal information will be

mentioned when the research is published.
