# Supplementary material for: Clinical Uncertainty Influences Antibiotic Prescribing for Upper Respiratory Tract Infections: A Qualitative Study of Township Hospital Physicians and Village Doctors in Rural Shandong Province, China
Source: Antibiotics (Basel). 2023 Jun 8;12(6):1027. doi: 10.3390/antibiotics12061027 (PMC10295071; doi:10.3390/antibiotics12061027)
Supplement: Supplementary file 1 [file antibiotics-12-01027-s001.zip › Supplementary Material 2-COREQ.pdf]

## Supplementary Material 2:

## Consolidated criteria for reporting qualitative studies (COREQ): 32-item checklist

| No Item | Guide questions/description | Reported on Page No. |
|---|---|---|
| **Domain 1: Research team and reflexivity**<br>**Personal Characteristics** | | |
| 1. Interviewer/facilitator | Which author/s conducted the interview or focus group? | Described at Page 3 |
| 2. Credentials | What were the researcher's credentials? E.g. PhD, MD | Described at Page 4 |
| 3. Occupation | What was their occupation at the time of the study? | Described at Page 4 |
| 4. Gender | Was the researcher male or female? | Female. |
| 5. Experience and training | What experience or training did the researcher have? | Described at Page 4 |
| **Relationship with participants** | | |
| 6. Relationship established | Was a relationship established prior to study commencement? | Described at Page 4 |
| 7. Participant knowledge of the interviewer | What did the participants know about the researcher? *e.g. personal goals, reasons for doing the research.* | Described at Page 4 |
| 8. Interviewer characteristics | What characteristics were reported about the interviewer/facilitator? *e.g. Bias, assumptions, reasons and interests in the research topic.* | Described at Page 5 |

| | | |
|---|---|---|
| **Domain 2: study design**<br>**Theoretical framework** | | |
| 9. Methodological orientation and Theory | What methodological orientation was stated to underpin the study? e.g. grounded theory, discourse analysis, ethnography, phenomenology, content analysis. | Described at Page 3 to 4 |
| **Participant selection** | | |
| 10. Sampling | How were participants selected? e.g. purposive, convenience, consecutive, snowball | Described at Page 3 |
| 11. Method of approach | How were participants approached? e.g. face-to-face, telephone, mail, email | Described at Page 3 |
| 12. Sample size | How many participants were in the study? | Described at Page 3 |
| 13. Non-participation | How many people refused to participate or dropped out? Reasons? | Not participants refused to participate. |
| **Setting** | | |
| 14. Setting of data collection | Where was the data collected? e.g. home, clinic, workplace | Described at Page 3 |
| 15. Presence of non-participants | Was anyone else present besides the participants and researchers? | Described at Page 3 |
| 16. Description of sample | What are the important characteristics of the sample? e.g. demographic data, date | Described at Page 5, Table 1. |
| **Data collection** | | |

| | | |
|---|---|---|
| 17. Interview guide | Were questions, prompts, guides provided by the authors? Was its pilot tested? | Described at Page 3 |
| 18. Repeat interviews | Were repeat interviews carried out? If yes, how many? | Not. |
| 19. Audio/visual recording | Did the research use audio or visual recording to collect the data? | Yes.<br>Described at Page 4 |
| 20. Field notes | Were field notes made during and/or after the interview or focus group? | Yes<br>Described at Page 4 |
| 21. Duration | What was the duration of the interviews or focus group? | Described at Page 4 |
| 22. Data saturation | Was data saturation discussed? | Yes.<br>Described at Page 3 |
| 23. Transcripts returned | Were transcripts returned to participants for comment and/or correction? | Yes.<br>Described at Page 4 |
| **Domain 3: analysis and findings**<br>**Data analysis** | | |
| 24. Number of data coders | How many data coders coded the data? | Two researchers coded the data.<br>Described at Page 4 |
| 25. Description of the coding tree | Did authors provide a description of the coding tree? | Described at Page 4 to 6 |
| 26. Derivation of themes | Were themes identified in advance or derived from the data? | Described at Page 5 |
| 27. Software | What software, if applicable, was used to manage the data? | Yes.<br>An iFLYTEK software (version SR502, iFLYTEK CO.LTD, China) was used to store the interview records. |

| | | |
|---|---|---|
| 28.Participant checking | Did participants provide feedback on the findings? | Yes.<br>Page 4 |
| **Reporting** | | |
| 29. Quotations presented | Were participant quotations presented to illustrate the themes / findings? Was each quotation identified? e.g. participant number | Yes.<br>Page 5 to 10 and Table 2 |
| 30. Data and findings consistent | Was there consistency between the data presented and the findings? | Page 5 to 10 and Table 2 |
| 31. Clarity of major themes | Were major themes clearly presented in the findings? | Page 5 |
| 32. Clarity of minor themes | Is there a description of diverse cases or discussion of minor themes? | Page 10 to 13 and Table 2 |

Developed from: Tong A, Sainsbury P, Craig J. Consolidated criteria for reporting qualitative research (COREQ): a 32-item checklist for interviews and focus groups. International Journal for Quality in Health Care. 2007. Volume 19, Number 6: pp. 349 – 357
